# Publisher Correction: Nitric oxide-dependent anaerobic ammonium oxidation

**DOI:** 10.1038/s41467-019-09797-4

**Published:** 2019-04-18

**Authors:** Ziye Hu, Hans J. C. T. Wessels, Theo van Alen, Mike S. M. Jetten, Boran Kartal

**Affiliations:** 1 Department of Microbiology, IWWR, Radboud University Nijmegen, Heyendaalseweg 135, 6525AJ Nijmegen, The Netherlands; 2 Translational Metabolic Laboratory, Department of Laboratory Medicine, Radboud University Medical Center, Geert Grooteplein-zuid 10, 6525GA Nijmegen, The Netherlands; 3 Present Address: Sanquin, Plesmanlaan 125, 1066 CX Amsterdam, The Netherlands; 4 Present Address: Microbial Physiology Group, Max Planck Institute for Marine Microbiology, Celsiusstraße 1, 28359 Bremen, Germany

**Keywords:** Bacterial physiology, Environmental microbiology

Correction to: *Nature Communications* 10.1038/s41467-019-09268-w, published online 18 March 2019

The original HTML version of this Article had an incorrect Published online date of 20 March 2019; it should have been 18 March 2019. This has been corrected in the HTML version of the Article. The PDF version was correct from the time of publication.

