# Author Correction: Nitric oxide-dependent anaerobic ammonium oxidation

**DOI:** 10.1038/s41467-022-30986-1

**Published:** 2022-06-07

**Authors:** Ziye Hu, Hans J. C. T. Wessels, Theo van Alen, Mike S. M. Jetten, Boran Kartal

**Affiliations:** 1 Department of Microbiology, IWWR, Radboud University Nijmegen, Heyendaalseweg 135, 6525AJ Nijmegen, The Netherlands; 2 Translational Metabolic Laboratory, Department of Laboratory Medicine, Radboud University Medical Center, Geert Grooteplein-zuid 10, 6525GA Nijmegen, The Netherlands; 3 Present Address: Sanquin, Plesmanlaan 125, 1066 CX Amsterdam, The Netherlands; 4 Present Address: Microbial Physiology Group, Max Planck Institute for Marine Microbiology, Celsiusstraße 1, 28359 Bremen, Germany

Correction to: *Nature Communications*; 10.1038/s41467-019-09268-w, published online 18 March 2019.

This Article contains the following errors in Figure 1:

The legend incorrectly reads “Empty triangles indicate nitrite concentration in the influent” and “Filled triangles indicate nitrate concentration in the effluent.” This should read “Filled triangles indicate nitrite concentration in the influent” and “Empty triangles indicate nitrate concentration in the effluent.”

In addition, nitrate concentration in the effluent in panel c is incorrectly indicated using filled triangles. The correct version of Fig. 1, showing empty triangles instead, is:
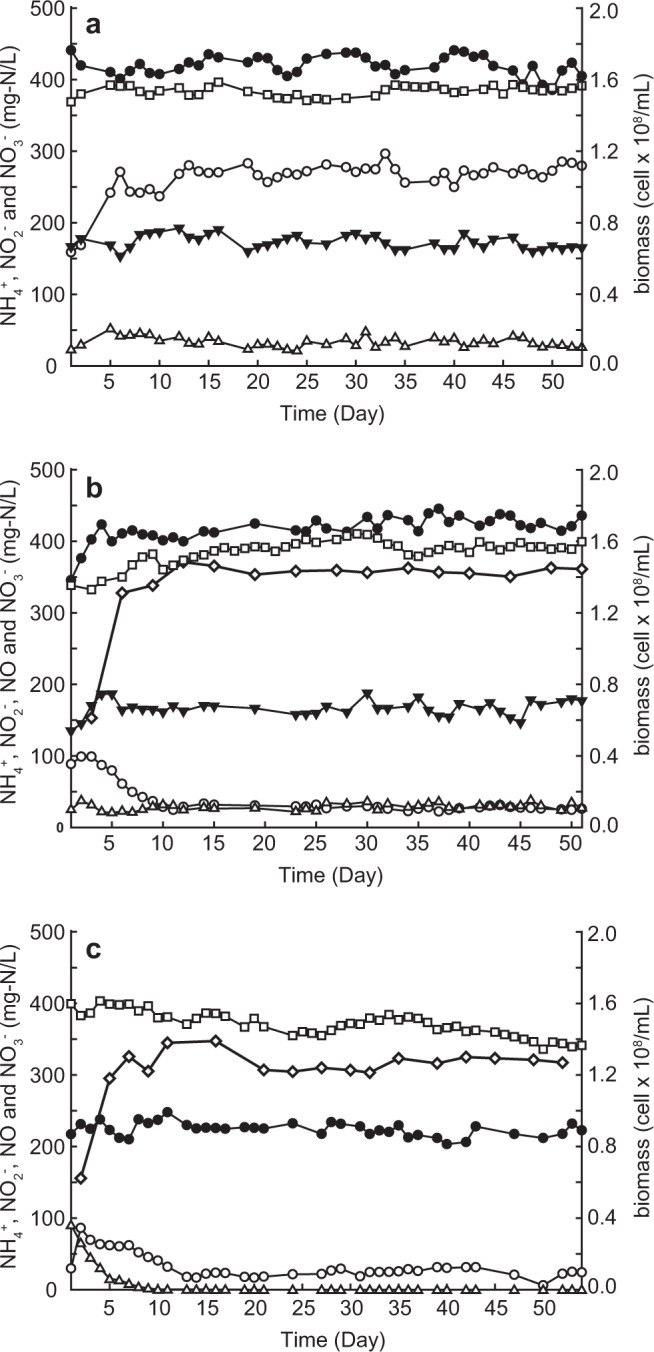


The errors have not been corrected in the PDF or HTML versions of the Article.

