# Supplementary material for: Nitric oxide-dependent anaerobic ammonium oxidation
Source: Nat Commun. 2019 Mar 18;10:1244. doi: 10.1038/s41467-019-09268-w (PMC6423088; doi:10.1038/s41467-019-09268-w)
Supplement: Supplementary file 2 — Description of Additional Supplementary Files [file 41467_2019_9268_MOESM2_ESM.pdf]

Description of Additional Supplementary Files

File Name: Supplementary Data 1
Description: Up-regulation of Kuenenia stuttgartiensis gene transcription in the reactor growing on NO and ammonium (Reactor III) compared to the reactor growing on nitrite and ammonium (Reactor I)

File Name: Supplementary Data 2
Description: Down-regulation of Kuenenia stuttgartiensis gene transcription in the reactor growing on NO and ammonium (Reactor III) compared to the reactor growing on nitrite and ammonium (Reactor I)
